# A search for non-chromosome 6 susceptibility loci contributing to rheumatoid arthritis

**DOI:** 10.1186/1753-6561-3-s7-s15

**Published:** 2009-12-15

**Authors:** Brian K Suarez, Robert Culverhouse, Carol H Jin, Anthony L Hinrichs

**Affiliations:** 1 Department of Psychiatry, Washington University School of Medicine, 660 South Euclid, Campus Box 8134, St. Louis, Missouri 63110, USA; 2 Department of Genetics, Washington University School of Medicine, 660 South Euclid, St. Louis, Missouri 63110, USA; 3 Department of Medicine, Washington University School of Medicine, 660 South Euclid, St. Louis, Missouri 63110, USA

## Abstract

We conducted a search for non-chromosome 6 genes that may increase risk for rheumatoid arthritis (RA). Our approach was to retrospectively ascertain three "extreme" subsamples from the North American Rheumatoid Arthritis Consortium. The three subsamples are: 1) RA cases who have two low-risk *HLA-DRB1 *alleles (*N *= 18), 2) RA cases who have two high-risk *HLA-DRB1 *alleles (*N *= 163), and 3) controls who have two low-risk *HLA-DRB1 *alleles (*N *= 652). We hypothesized that since Group 1's RA was likely due to non-HLA related risk factors, and because Group 3, by definition, is unaffected, comparing Group 1 with Group 2 and Group 1 with Group 3 would result in the identification of candidate susceptibility loci located outside of the MHC region. Accordingly, we restricted our search to the 21 non-chromosome 6 autosomes. The case-case comparison of Groups 1 and 2 resulted in the identification of 17 SNPs with allele frequencies that differed at *p *< 0.0001. The case-control comparison of Groups 1 and 3 identified 23 SNPs that differed in allele frequency at *p *< 0.0001. Eight of these SNPs (rs10498105, rs2398966, rs7664880, rs7447161, rs2793471, rs2611279, rs7967594, and rs742605) were common to both lists.

## Background

Rheumatoid arthritis (RA) is a chronic inflammatory disorder in which the articular joints are gradually destroyed. Occasionally there is systemic involvement, which can include pulmonary fibrosis and vasculitis in various organs. The etiology of RA is complex, with significant genetic and environmental components.

Among the genetic components, genes in the MHC region on chromosome 6p21.3 are widely acknowledged to be the major player, with the *HLA-DRB1 *locus as the leading suspect [1,2]. Five alleles dramatically increase risk (DRB1*0401, *0404, *0405, *0408, and *0409), while other alleles appear to confer a moderate increase in risk (DRB1*0101, *0102, *0104, *0105, *1001, *1402, and *1406). In what follows we denote the high-risk alleles as H, the moderate risk alleles as M and the low-risk alleles as L. Most of the high-risk alleles possess a shared epitope (SE) of five amino acids at positions 70-74 in the third hypervariable region of the DRβ1chain [3]. Genotypes consisting of alleles *0401/DRX (where *DRX *denotes a non-SE allele) and *0404/DRX are estimated to increase the relative risk of RA by 4.7-fold and 5.0-fold, respectively, while the *0401/*0401 genotype carries a relative risk of 18.8 and the compound heterozygote *0401/*0404 has a relative risk of 31.0 [4,5]. We included in the high-risk group individuals whose genotype was reported as *4/4 *or 4/*0401.

Not all persons with RA possess one or two high-risk SE alleles, however. It is estimated that in persons of European ancestry, approximately 30% do not carry an SE-encoding allele [2]. In the data made available to the Genetic Analysis Workshop 16 participants, the percentage of cases carrying two low risk *DRB1 *alleles is much smaller than 30%. We hypothesize that RA cases with two non-SE encoding *DRB1 *alleles constitute a subgroup of patients that are enriched for other susceptibility alleles located elsewhere in the genome. Accordingly, we undertook a genome-wide association (GWA) analysis that compares single-nucleotide polymorphism (SNP) allele frequencies in two groups of cases-those whose *DRB1 *genotype contains two high-risk alleles (HH) and those whose *DRB1 *genotype contains two low-risk alleles (LL)-and one group of controls whose *DRB1 *genotype also consists of two LL alleles.

## Methods

Table 1 reports the distribution of *HLA-DRB1 *genotypes in cases and controls from the North American Rheumatoid Arthritis Consortium (NARAC). There were an additional 69 cases and a single control for whom *DRB1 *genotypes were not available. Table 1 underscores the substantial difference between cases and controls at DRB1 (χ^2 ^= 772.2, *p *= 1.2 × 10^-164^). The table also shows that in the NARAC sample there are very few cases with the low-risk LL genotype (2.25%). Because of the very small LL case sample, we chose to use Fisher's exact test for all comparisons, realizing the statistic is severely conservative when interpreted with reference to conventional alpha levels [6]. Also, because of the small size of the LL case sample, and because some SNPs will have dropped genotypes, we required that at least 15 of the 18 LL cases be genotyped. Because we categorized the high- and low-risk subgroups according to their *HLA-DRB1 *genotypes, we restrict our attention to the 21 non-chromosome 6 autosomes.

**Table 1:** Sample size for various subdivisions of the NARAC data

	SS			SN		NN	
				
	HH	HM	MM	HL	ML	LL	Total
Case	163	156	15	322	125	18	799
Controls	22	34	21	249	215	652	1193

^a^Moderate and high risk *DRB1 *alleles are denoted by "S" and low risk alleles by "N"

There is some evidence for population substructure in this sample-perhaps occasioned by the fact that patients were drawn from rheumatology clinics across North America, while all of the controls were selected from participants who were part of the New York Cancer Project [7]. We searched for systematic differences in the clinical variables that might distinguish the two case subsamples we compared in this study. We also undertook an analysis of substructure for the two case samples based on SNPs. We selected every 50^th ^SNP from all of the autosomes, except chromosome 6, for a sample of 9,920 SNPs, and performed an EIGENSTRAT analysis [8].

## Results

Table 2 reports the comparison of the few clinical variables that were included with the case dataset. With respect to sex ratio, only one of the 18 (5.6%) LL cases is male, whereas 53 of the 163 HH cases (32.5%) are male (*p *= 0.01). Despite the unequal variances, no differences were seen in the mean values for the two continuous clinical variables anti-cyclic citrullinated peptide (anti-CCP) and rheumatoid factor IgM titers. Unfortunately, no data were made available for an important behavioral/environmental variable, namely smoking.

**Table 2:** Distribution of three clinical variables in high- and low-risk cases

	Mean (± SD)		
		
	High-risk cases	Low-risk cases	*p*-Value
Sex ratio (Male:female)	32.5%	5.6%	0.01
Anti-CCP	195.4 (246.8)	210.1 (162.4)	0.74
Rheumatoid factor	284.8 (438.1)	546.0 (927.1)	0.25

Table 3 reports the results for the two GWA analyses. The comparison of the LL and HH cases resulted in the identification of 17 SNPs with allele frequencies that differed at *p *< 0.0001. The comparison of the LL cases and the LL controls resulted in the identification of 23 SNPs. Eight SNPs are common to both lists. Also reported in Table 3 are the minor allele frequencies (MAF) in the LL cases and the frequency of the same allele (which may be the majority allele) in the HH cases and LL controls, as well as the closest known gene or predicted gene.

**Table 3:** Results of the GWA analyses for SNPs that differed between the comparison groups at *p *< 0.0001: minor allele frequency in LL cases

SNP	LL cases	HH cases	LL controls	Physically closes gene
LL cases vs. HH cases				
**rs10498105^a^**	**0.306**	**0.66**	**-**	***EPHA4***
**rs2398966**	**0.111**	**0.469**	**-**	***AC012154.16***
rs2293004	0.361	0.699	-	*AADAC*
**rs7664880**	**0.361**	**0.704**	**-**	***AC096566.2***
rs1845344	0.389	0.727	-	*MAD2L1*
**rs7447161**	**0.083**	**0.396**	**-**	***FER***
rs1350309	0.028	0.298	-	*AC019176.4*
rs3731239	0.333	0.687	-	*CDKN2A*
rs10813797	0.278	0.625	-	*ACO1*
rs7865082	0.361	0.713	-	*DDX58*
**rs2793471**	**0.222**	**0.564**	**-**	***AL135933.11***
**rs2611279**	**0.111**	**0.426**	**-**	***SLC6A15***
**rs7967594**	**0.111**	**0.426**	**-**	***SLC6A15***
rs2148443	0.139	0.497	-	*TMFRSF19*
rs2567506	0.111	0.429	-	*SLC39A11*
**rs742605**	**0.111**	**0.436**	**-**	***TRIB3***
rs5771716	0.389	0.724	-	*FAM19A5*
				
LL cases vs. LL cases				
rs11684785	0.094	-^b^	0.423	*TMEM163*
**rs10498105**	**0.306**	**-**	**0.686**	***EPHA4***
**rs2398966**	**0.111**	**-**	**0.413**	***AC012154.16***
**rs7664880**	**0.361**	**-**	**0.684**	***AC096566.2***
rs173948	0.222	-	0.548	*TRIO*
rs42404	0.083	-	0.386	*TRIO*
rs890937	0.083	-	0.377	*TRIO*
rs358753	0.111	-	0.421	*TRIO*
rs27761	0.139	-	0.452	*TRIO*
rs27114	0.111	-	0.442	*TRIO*
rs40066	0.389	-	0.707	*FBXL17*
rs40065	0.389	-	0.707	*FBXL17*
**rs7447161**	**0.083**	**-**	**0.376**	***FER***
rs921634	0.028	-	0.299	*PKD1L1*
rs1324208	0.389	-	0.719	*SMARCA2*
rs1396553	0.417	-	0.747	*SMC3*
**rs2793471**	**0.222**	**-**	**0.584**	***AL135933.11***
rs6539845	0.139	-	0.452	*SLC6A15*
rs11116408	0.139	-	0.457	*SLC6A15*
**rs2611279**	**0.111**	**-**	**0.446**	***SLC6A15***
**rs7967594**	**0.111**	**-**	**0.447**	***SLC6A15***
rs1380412	0.111	-	0.438	*SLC6A15*
**rs742605**	**0.111**	**-**	**0.419**	***TRIB3***

^a^SNPs common to both lists are boldface.

^b^-, not applicable.

**Figure 1:**
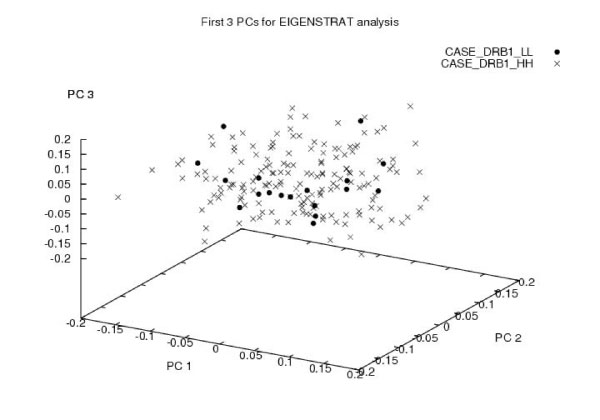
**Plot of the first three principal components from the EIGENSTRAT analysis showing the distribution of the LL and HH cases**. No obvious substructure is apparent.

Figure 1 plots the distribution of the LL and HH cases for the first three principal components from the EIGENSTRAT analysis. Visual inspection gives no evidence that the two groups of cases differ in any systematic fashion. Neither does the distribution of eigenvalues. When the low risk controls are added, however, both visual inspection and the distribution of eigenvalues reveals that one component is required to adjust for stratification within the sample.

## Discussion

We are, of course, aware that a sample of *N *= 18 cases is extremely small by today's standards. Nonetheless, we are mindful that even small samples can be useful if the genetic effects are large enough. For instance, Cudworth and Woodrow [9] were able to confirm the involvement of the MHC in type I diabetes using a sample of only 17 affected sib-pair families. Moreover, many of the early gene expression studies employed very small sample sizes, yet provided useful insights into various expression patterns.

Although it is customary to report SNP allele frequencies in terms of the MAF (as we did for the LL cases in Table 3), our hypothesis predicts higher allele frequencies for non-chromosome 6 susceptibility genes in RA subjects who have LL genotypes at the HLA-DRB1 locus. For all of the SNPs listed in Table 3, the MAF in the LL cases are less than the comparison group. Accordingly, we would predict that the opposite SNP allele in the LL cases is probably in linkage disequilibrium with alleles at a functional gene.

Some of the SNPs listed in Table 3 are either in the same gene (e.g., *TRIO*, where all of the listed SNPs are intronic) or are close to the same gene (e.g., *SLC6A15 *and *FBXL17*). We carried out an analysis of linkage disequilibrium and estimated the *r*^2 ^values between these adjacent SNPs from the NARAC control panel and obtained estimates of *r*^2 ^= 1.0 for the two SNPs near *FBXL17*, *r*^2 ^= 0.384, 0.949, 0.821, 0.801, and 0.734 for the five pairs of adjacent SNPs from *TRIO *(from rs173948 to rs27114), and *r*^2 ^= 0.975, 0.837, 0.997, and 0.960 for the four pairs of adjacent SNPs near *SLC6A15*.

Table 4 summarizes non-chromosome 6 loci/regions that have been identified by others as contributing to the risk of RA. None of the SNPs identified in this study lie within 100 kb of any of these loci/regions listed in Table 4.

**Table 4:** Previously identified non-chromosome 6 loci or regions that contribute to the risk of RA

Locus	Location	Type of variation	Reference
*SLC22A4*	5q31	Intronic SNP	Tokuhiro et al. [10]
*RUNX1*	21q22.3	Intronic SNP	Tokuhiro et al. [10]
*PADI4*	1p36.13	Production of RA-specific autoantibodies	Suzuki et al. [11]
*PTPN22*	1p13	Non-synonymous coding SNP	Begovich et al. [12]
*MHC2TA*	16p13	SNP in the type III of the MHC class II transactivator	Swanberg et al. [13]
*STAT4*	2q32.2-3	Intronic SNP	Remmers et al. [14]
*TRAF1-C5*	9q33-34	A 100-kb region	Plenge et al. [7]

## Conclusion

We carried out a GWA study using an extreme sampling design on three subsets of the NARAC data. We identified 17 SNPs and 23 SNPs that distinguished the LL cases from the HH cases, and the LL cases from the LL controls at *p *< 0.0001, respectively. Eight SNPs are common to both lists, although the presence of significant linkage disequilibrium suggests that the actual overlap would be less. We used a variety of sources to identify the nearest gene, or predicted gene [15-19]. For some of these SNPs, however, there are multiple genes in their vicinity.

## List of abbreviations used

GWA: genome wide association; H: High-risk allele; L: Low-risk allele; M: Moderate-risk allele; MAF: Minor allele frequency; NARAC: North American Rheumatoid Arthritis Consortium; RA: Rheumatoid arthritis; SE: Shared epitope; SNP: Single-nucleotide polymorphism.

## Competing interests

The authors declare that they have no competing interests.

## Authors' contributions

BKS conceived and designed the study and drafted the manuscript. All of the authors participated equally in managing the data and in the statistical analysis of the data.
